# Housing Instability and HIV Risk: Expanding our Understanding of the Impact of Eviction and Other Landlord-Related Forced Moves

**DOI:** 10.1007/s10461-020-03121-8

**Published:** 2021-01-02

**Authors:** Allison K. Groves, Linda M. Niccolai, Danya E. Keene, Alana Rosenberg, Penelope Schlesinger, Kim M. Blankenship

**Affiliations:** 1 Department of Community Health and Prevention, Drexel University Dornsife School of Public Health, Nesbitt Hall, 3215 Market Street 416, Philadelphia, PA 19140 USA; 2 Department of Epidemiology of Microbial Diseases, Yale University School of Public Health, 60 College Street, New Haven, CT 06510 USA; 3 Department of Social and Behavioral Sciences, Yale University School of Public Health, 60 College Street, New Haven, CT 06510 USA; 4 Department of Sociology, American University, 4400 Massachusetts Avenue, Washington DC, 20016 USA

**Keywords:** Eviction, Housing, Sexual risk behavior, HIV/AIDS

## Abstract

The study purpose is to comprehensively measure landlord-related forced moves (inclusive of, but not restricted to, legal eviction), and to examine whether landlord-related forced moves is associated with HIV risk. Baseline survey data was collected between 2017 and 2018 among 360 low-income participants in New Haven, Connecticut. We used multivariable logistic regression analyses to examine associations between landlord-related forced moves and HIV sexual risk outcomes. Seventy seven out of three hundred and sixty participants reported a landlord-related forced move in the past 2 years, of whom 19% reported formal eviction, 56% reported informal eviction and 25% reported both. Landlord-related forced moves were associated with higher odds of unprotected sex (AOR 1.98), concurrent sex (AOR 1.94), selling sex for money or drugs (AOR 3.28), exchange of sex for a place to live (AOR 3.29), and an HIV sexual risk composite (ARR 1.46) (p < .05 for all). We found robust associations between landlord-related forced moves and HIV sexual risk. Findings suggest that the social and economic consequences of landlord-related forced moves may impact sexual vulnerability.

## Introduction

More than 2 million low-income individuals and families in the United States are legally evicted from their housing every year [1], which has potentially devastating impacts on their health, well-being, and future housing stability [2]. Most legal evictions occur because of non-payment of rent, which is primarily a result of high housing costs [3]. Housing costs have risen significantly faster than income throughout the United States; nearly three quarters of extremely low-income renter households spend more than half of their income on rent and utilities [4]. As such, any unanticipated emergency or serious illness (e.g., job loss, medical bills, COVID-19, etc.) can decrease individuals’ ability to pay their monthly rent and increase their risk of eviction.

While there is a growing literature on the prevalence and health impacts of eviction [5–14], most of these studies focus on the health impacts of legal eviction [8–14]. This likely results in an underestimate of the prevalence of low-income individuals who are forcibly removed from their housing each year. Evidence suggests that renters may be forced by landlords to move through less formal processes. For example, a landlord may tell a tenant they have to move, raise the rent, and/or have the property foreclosed [15–17]. Indeed, Desmond and Shollenberger highlight how a narrow definition of eviction underestimates the prevalence of forced moves among urban renters in Milwaukee [16]. Specifically, they found that one in eight renters experienced a landlord-related forced move in the past two years, of which only 24% were the result of a legal eviction. To our knowledge, no other studies have examined the prevalence of landlord-related forced moves conceptualized more broadly.

Not only is there a gap in understanding the prevalence of landlord-related forced moves, there is also limited literature on the impacts of such moves on health. One health outcome that may be sensitive to the impacts of forced moves is HIV. Theoretically, the pathways through which legal eviction and landlord-related forced moves impact HIV sexual risk should be similar, given that both constitute disruptions in housing. Such disruptions likely have significant psychological consequences [13] related to increased individual stress [18, 19] and reduced mental bandwidth for decision-making [20]. As a result of the disruptions of a forced move, individuals may be more focused on addressing day-to-day survival needs and be less future-oriented [21], may have lower self-efficacy [22], and/or may have decreased positive coping skills [23], each of which might negatively impact their ability to engage in health-promoting HIV prevention behaviors, such as condom use. Furthermore, the stress or trauma associated with an adverse event such as a forced move may impact power dynamics within relationships and have negative implications for an individual’s ability to negotiate and use condoms in sexual encounters [24]. Forced moves may also contribute to the fragmenting of social networks, and specifically, may disrupt or prevent relationship stability, which may lead to higher levels of concurrent, or overlapping, sexual partnerships [12]. And, the economic consequences of a forced move may increase the likelihood of transactional sex, including the exchange of sex for a place to live [12, 25].

Therefore, the purpose of the current study is to expand understanding of the impact of being forced to move on HIV by comprehensively measuring the extent of landlord-related forced moves (including legal evictions), and to examine whether these moves are associated with HIV sexual risk among low-income individuals living in racially segregated neighborhoods.

## Methods

### Study Design and Sample

Data for these analyses were collected as part of the Justice Housing and Health Study (JustHouHS), a longitudinal study that focuses on understanding the intersection of mass incarceration and housing vulnerabilities and their impact on HIV risk. The study was conducted in the city of New Haven, CT. New Haven’s legal eviction rate is over 4%, which is the 69th highest eviction rate of large cities in the nation and also one of the highest eviction rates among cities in New England [26].

Participants were recruited into the study using a combination of flyers, outreach to local service providers, community meetings, and snowball sampling. Research staff screened 616 individuals, of whom 145 were not eligible and 71 did not enroll. Participants were screened by telephone. Those who did not have access to a phone were screened in person. Participants were eligible for JustHouHS if (1) they were 18 years or older and a resident of New Haven, (2) no household members were already in the study, and (3) they met one of the following criteria: (a) received housing or food assistance in the past year, (b) were Medicaid recipients, (c) were homeless, or (d) resided in low-income census tracts (i.e., more than 20% of residents lived below the federal poverty level). The sample was also stratified to include 200 individuals released from prison or jail in the past year and 200 who were not recently released (though may have had a history of incarceration).

All eligible individuals provided consent to participate and then completed a self-administered, computer-assisted survey, which took between 1 and 2 h to complete. Individuals received a $50 gift card after completing the survey. The study protocol was approved by the IRB at Yale University.

A total of 400 participants enrolled in the study. For this analysis, we excluded participants who reported being HIV positive (n = 37) or who were missing data (n = 3) for a total analytic sample of 360 participants.

## Measures

### Landlord-Related Forced Moves

Participants were coded as having a landlord-related forced move if: (1) they reported that they had been officially evicted in the past two years; and/or (2) they reported that they had moved in the past 2 years for any of the following reasons: I was evicted, the landlord raised the rent, I was forced to move because of non-payment of rent, I was forced to move because of damage to rental unit, I was forced to move because I was accused of illegal drug activities (including sale and/or use), I was forced to move because I was accused of illegal activity (not drug related), I was forced to move because landlord said there were too many people living there, I was forced to move because the landlord went into foreclosure.

### HIV Sexual Risk Outcomes

The outcomes examined in this analysis are based on measures that have been commonly used to examine HIV and/or STI risk in other studies (e.g., [27–34]). All participants were asked if they had had any unpaid sexual partners in the past six months. If they responded “yes”, they then answered a series of questions about their sexual behavior with each of these partners for up to four sexual partners. (1) *Unprotected sex* Participants reported how often they used condoms with each sexual partner they had either vaginal or anal sex with in the past six months. Individuals who reported “always using condoms” or who reported “no sexual partners” in the past six months received a ‘0′ for unprotected sex; all others received a ‘1′. (2) *Concurrent sex across partners* Participants were asked the following question for each sexual partner: “during the same time period that you were having sex with [partner 1], in the last 6 months, were you also having sex with anyone else?” Individuals who responded “yes” to this item for any sexual partner received a ‘1′; individuals who responded “no” to this item for each sexual partner or who reported no sexual partners received a ‘0′. (3) *Perceived partner concurrency* Participants reported whether they perceived each partner they listed also had other sexual partners in the past six months. Individuals who suspected that any partner had another sexual partner received a ‘1;’ individuals who did not suspect that their partner had other sexual partners or reported no sexual partners in the last 6 months received a ‘0′. In addition to partner-specific questions, participants were asked if they had (4) *Sold sex in exchange for money or drugs in the last 6 months* Individuals who responded “no” received a ‘0′; all others received a ‘1′. Finally, in a series of questions about their housing arrangements, participants were asked if, in the last 6 months, they had provided something other than money in exchange for a place to stay. Those who responded “yes” were asked if they had (5) *Provided sex in exchange for a place to live in the last six months* Individuals who reported “no” received a ‘0′; all others received a ‘1.’ (6) *Composite HIV sexual risk index* The composite HIV sexual risk index is a sum of all prior outcomes described. If participants reported zero sexual risks, they received a ‘0′. The composite measure captures sexual risk exposure in a way that the specific binary measures do not. That is, any one of the sexual risks in and of themselves become amplified as risks in coordination with the other(s). For example, someone who is not using condoms but is not having sex with anyone else, and whose partner is not having sex with anyone else, is deemed at lower risk in the composite index. On the other hand, someone who is not using condoms and is also engaged in other risky behaviors receives a higher risk score. Conceptually, aggregating multiple risk indicators to capture behaviors occurring within and across sexual partnerships may more accurately reflect HIV sexual risk than any single measure.

### Covariates

We included the following sociodemographic variables to describe our sample: age, gender (male, female), race/ethnicity (black, white, other), education (less than high school, high school, more than high school), worked in the past 6 months (yes/no), individual income in the last month (reported in dollars). We also examine the following variables as potential confounders: currently homeless (yes/no), currently receiving housing assistance (yes/no), ever had a mental health diagnosis (yes/no), drug use in the past 30 days (yes/no), injection drug use in the past 30 days (yes/no), heavy alcohol use in the past 30 days (drank more than 15 days in the past 30 days (yes/no), and incarceration in the past two years (yes/no), which was verified using data from the Connecticut Judicial Branch.

### Statistical Analyses

First, we examined sociodemographic differences between participants with and without a landlord-related forced move using Pearson’s chi-square tests for categorical variables and t-tests for continuous variables. Next, we conducted bivariate (model 1) and multivariate (model 2) logistic regression models (for unprotected sex, concurrent sex, perceived partner concurrency, sold sex in exchange for money/drugs, and provided sex in exchange for a place to live) and Poisson regression models (for the composite HIV sexual risk variable) to examine the association between a landlord-related forced move and each of our six HIV sexual risk outcomes. Odds ratios and risk ratios, along with their corresponding 95% confidence intervals, were estimated using logistic and Poisson regression models, respectively. Two-sided type I error < 0.05 was used in all analyses. All statistical analysis was performed using SAS software, version 9.4 [35]. In multivariate analyses, we controlled for sociodemographic variables (age, gender, race/ethnicity, education, income) and variables that we theorized might be confounders that were significant in bivariate analysis at the p = 0.10 level (i.e., homelessness, mental health diagnosis, drug use).

In addition, we did sensitivity analysis to examine the robustness of our findings. First, we examined whether our results changed based on how we conceptualized the landlord-related forced moves variable. Specifically, we examined whether the inclusion/exclusion of the following items affected the findings: I was accused of illegal drug activities, I was accused of illegal activity, and the landlord raised the rent. Second, we examined whether our results changed if we assessed HIV sexual risk outcomes among only those who reported sex in the last six months. We also examined whether the findings changed if we used a more conservative HIV sexual risk composite (i.e., one that did not include those who reported exchanging sex for a place to live) to account for the possibility that someone who exchanges sex for a place to live might also have unsafe sex with that same partner that they are living with, since it is not clear whether these are distinctive behaviors that might amplify risk. There were no substantive changes in the pattern of results for any of the sensitivity analyses.

## Results

The mean age of participants was 44.0 years (SD 11.5) and our sample was predominantly male (69%) (Table 1). Only 4% of men reported having sex with men. Nearly two-thirds of participants identified as African American (63%). Almost three-quarters had a high school education or lower (71%). Half of the sample reported working in the past 6 months, with a mean monthly income of $889.51.

**Table 1:** Participant characteristics at baseline

	All participants (N = 360)		Forced move (N = 77)		Not forced move (N = 283)		Test statistic	Difference between groups
	N	%	N	%	N	%		P-value
Age								
Years (mean, SD)	44.02 (11.54)		41.99 (12.33)		44.57 (11.27)		*t*(358) = 1.75	0.08
Gender								
Male	250	69	48	62	202	71	χ(1)^2^ = 2.33	0.13
Female	110	31	29	38	81	29		
Race								
Black	225	63	51	66	174	61	χ(2)^2^ = 1.14	0.56
White	101	28	21	27	80	28		
Other	34	9	5	6	29	10		
Education								
Less than HS	81	23	12	16	69	24	χ(2)^2^ = 3.78	0.15
HS	174	48	44	57	130	46		
More than HS	105	29	21	27	84	30		
Individual income past month								
Dollars (mean, SD)	889.51 (5302.10)		751.10 (2499.40)		927.20 (5839.40)		*t*(358) = 0.39	0.70
Worked in past 6 months								
Yes	181	50	40	52	141	50	χ(1)^2^ = 0.11	0.74
No	179	50	37	48	142	50		
Recent drug use (30 days)								
Yes	99	28	27	35	72	25	χ(1)^2^ = 2.81	0.09
No	261	73	50	65	211	75		
Recent injection drug use (30 days)								
Yes	13	4	5	6	8	3	χ(1)^2^ = 2.34	0.13
No	347	96	72	94	275	97		
Recent heavy alcohol use (30 days)								
Yes	38	11	12	16	26	9	χ(1)^2^ = 2.62	0.11
No	322	89	65	84	257	91		
Recent incarceration (past 2 years)								
Yes	197	55	44	57	153	54	χ(1)^2^ = 0.23	0.63
No	163	45	33	43	130	46		
Have a mental health diagnosis								
Yes	196	54	51	66	145	51	χ(1)^2^ = 5.49	0.02
No	164	46	26	34	138	49		
Currently receiving housing assistance								
Yes	63	18	12	16	51	18	χ(1)^2^ = 0.25	0.62
No	297	83	65	84	232	82		
Recent homelessness (past 6 months)								
Yes	121	34	38	49	83	29	χ(1)^2^ = 10.87	0.001
No	239	66	39	51	200	71		

More than one in five individuals reported a landlord-related forced move in the past 2 years (21%, n = 77/360; Table 2). Of these, 19% reported experiencing a formal eviction only (n = 15/77), 56% reported another type of landlord-related forced move (n = 43/77) and 25% reported both (n = 19/77).

**Table 2:** Prevalence of forced moves

Legally evicted	Frequency (n)	Proportion (%)
Not legally evicted in the past 2 years	326	91
Legally evicted in the past 2 years	34	9

Other landlord-related forced moves (responded yes to at least one of the following: I was forced to move in the last 2 years because…)^a^	Frequency (n)	
I was evicted	31	
landlord raised the rent	8	
of non-payment of rent	22	
damage to the rental unit	1	
I was accused of illegal drug activities (including sale and/or use)	11	
I was accused of illegal activity (not drug related)	6	
the landlord said there were too many people living there	13	
the landlord went into foreclosure	8	

Landlord-related forced move (binary)		
No landlord-related forced move	298	83
Landlord-related forced move	62	17

Forced move^b^		
Not legally evicted or forced to move by landlord	283	79
Legally evicted or landlord-related forced move	77	21

^a^Check all that apply

^b^15 were only evicted, 43 only reported a landlord-related forced move, 19 responded 'yes' to both questions

As seen in Table 3, slightly more than half of all participants reported unprotected sex in the past six months (58%), one in five reported concurrent sex (21%), and one in four had the perception that their partner was engaging in concurrent sex (26%). Fewer than one in ten provided sex in exchange for a place to live (4%) or sold sex for money or drugs (8%). Finally, 38% of individuals had zero risk on the HIV sexual risk composite, 34% reported one risk, and the remainder reported two or more risks.

**Table 3:** Prevalence of HIV sexual risk behaviors

Condom use	Frequency (n)	Proportion (%)
Unprotected sex	210	58
Consistent condom use/no sex	150	42
Concurrent sex		
Engages in concurrent sex	76	21
Not concurrent/no sex	284	79
Perceived concurrent sex		
Perceived partner engages in concurrent sex	93	26
Not concurrent/no sex	267	74
Transactional sex		
Exchanged sex for money or drugs	28	8
No exchange/no sex for money or drugs	332	92
Provided sex in exchange for a place to stay	16	4
No exchange/no sex	344	96
Composite HIV sexual risk		
0	135	38
1	121	34
2	35	10
3	50	14
4	13	4
5	6	2

A few theoretically relevant covariates were marginally associated with reporting a landlord-related forced move (Table 1). Specifically, while 54% of participants in the sample reported a mental health diagnosis, mental health diagnosis was higher among individuals reporting a landlord-related forced move (66% vs. 51%, χ(1)^2^ = 5.49, p = 0.02). Similarly, recent homelessness was higher among individuals reporting a landlord-related forced move (49% vs 29%, χ(1)^2^ = 10.87, p = 0.001). Further, while nearly one-third of all participants reported recent drug use, drug use was marginally higher among individuals reporting a landlord-related forced move (34% vs. 25%, χ(1)^2^ = 2.81, p = 0.09). On the other hand, landlord-related forced moves did not vary based on an individual’s incarceration history, receipt of housing assistance, or other measures of substance use (i.e., heavy alcohol use or injection drug use). Finally, while younger individuals were marginally more likely to report a landlord-related forced move (41.99 vs 44.57, *t*(358) = 1.75, p = 0.08), there were no other differences in landlord-related forced moves by gender, race/ethnicity, education, work, or income.

As seen in Table 4, having experienced a landlord-related forced move was consistently associated with HIV sexual risk in both bivariate and multivariate models across most of the outcomes. Specifically, the odds of unprotected sex were significantly higher among individuals reporting a landlord-related forced move (Odds ratio [OR] 2.23, 95% CI 1.28–3.89; Adjusted odds ratio [AOR] 1.98, 95% CI 1.09–3.59). Similarly, the odds of reporting concurrent sex were also significantly higher for individuals reporting a landlord-related forced move (OR 2.38, 95% CI 1.35–4.17; AOR 1.94, 95% CI: 1.05–3.60). In bivariate analysis, the odds of perceived partner concurrency were marginally higher for individuals reporting a landlord-related forced move as compared to individuals not reporting a landlord-related forced move, but results were attenuated in multivariate analysis (OR 1.64, 95% CI 0.95–2.84; AOR 1.38, 95% CI 0.76–2.49). The odds of selling sex for money or drugs were significantly higher for individuals reporting a landlord-related forced move (OR 3.63, 95% CI 1.64–8.01; AOR 3.28, 95% CI 1.35–7.95), as were the odds of providing sex in exchange for a place to live (OR 5.22, 95% CI 1.87–14.51; AOR 3.29, 95% CI 1.08–10.04). Finally, a landlord-related forced move was also positively associated with the composite HIV sexual risk composite (Risk ratio [RR] 1.63, 95% CI 1.33–2.00; Adjusted risk ratio [ARR] 1.46, 95% CI 1.18–1.81).

**Table 4:** Bivariate and multivariate associations between forced move and HIV sexual risk

	Model 1: bivariate associations				Model 2: multivariate associations^a^			
	OR^b^	95% CI	Wald χ^2^	p-value	AOR^b^	95% CI	Wald χ^2^	p-value
Unprotected sex in last 6 months	2.23	1.28–3.89	8.10	0.004	1.98	1.09–3.59	32.57	0.02
Concurrent sex in last 6 months	2.38	1.35–4.17	9.09	0.003	1.94	1.05–3.60	33.67	0.03
Perceived partner has concurrent sex in last 6 months	1.64	.95–2.84	3.18	0.07	1.38	.76–2.49	28.11	0.29
Provided sex in exchange for a place to live in the last 6 months	5.22	1.87–14.51	10.03	0.002	3.29	1.08–10.04	20.17	0.04
Sold sex for money or drugs in the last 6 months	3.63	1.64–8.01	10.20	0.001	3.28	1.35–7.95	29.43	0.009

	RR^c^	95% CI	Pearson χ^2^	p-value	ARR^c^	95% CI	Pearson χ^2^	p-value
Composite HIV sexual risk	1.63	1.33–2.00	466.81	< 0.0001	1.46	1.18–1.81	433.35	0.0005

*OR* odds ratio, *95% CI* 95% confidence intervals, *RR* risk ratio

^a^Controlled for age, gender, race/ethnicity, education, income, recent drug use, homelessness, mental health diagnosis

^b^Modeled using logistic regression models

^c^Modeled using Poisson regression models

## Discussion

Attention to the relevance and health impacts of eviction for poor Americans has increased in recent years [11, 13]. However, there is a gap in our knowledge regarding both the extent of landlord-related forced moves (that are inclusive of, but not restricted to, legal evictions) and the impact of landlord-related forced moves on HIV risk. In the current study, we examined the prevalence of landlord-related forced moves (both through legal eviction and other types of landlord-related forced moves) and whether the disruption of a landlord-related forced move was associated with HIV sexual risk among a sample of low-income residents in New Haven, Connecticut. We found that landlord-related forced moves were common for our participants. Furthermore, consistent with expectations, we found robust associations between landlord-related forced moves and HIV sexual risk across a broad range of outcomes. We elaborate on key findings and their implications below.

Approximately one in five participants (21%) reported a landlord-related forced move in the past two years. Similar to existing literature [16], we found that legal eviction represented only a portion of those who experienced a landlord-related forced move. The findings serve as a reminder that a narrow focus on eviction fails to capture the broad ways in which landlords may exert power over their tenant(s). This is of critical importance in a housing scarce environment in which many low-income tenants have limited negotiating power [17]. Collectively, the findings from this growing body of research point to the importance of broadening conceptualization of landlord-related forced moves so that we may fully understand not only the scope of the problem but also its impacts.

Furthermore, our findings suggest that the social and economic impacts of landlord-related forced moves may have consequences for sexual vulnerability. That is, as hypothesized, we found that forced moves were consistently and positively associated with sexual risk across all but one outcome. Specifically, individuals who experienced a landlord-related forced move were more likely to report unprotected sex, concurrent sex, and transactional sex (i.e., providing sex for money, drugs, or housing). There are multiple pathways through which forced moves might impact these sexual practices [12]. For example, the stress of a landlord-related forced move may decrease psychological well-being and/or negatively impact negotiating power within a relationship, thereby affecting one’s ability to use and/or interest in using protection when having sex. A landlord-related forced move may also negatively impact partnership stability, which may in turn lead an individual to seek out housing with someone else who might become a sexual partner out of necessity. This may increase their likelihood of reporting multiple partners (concurrency) and/or exchanging sex for a place to stay. Future research might examine whether psychological well-being and social network disruptions are explanatory mediators of the association between landlord-related forced moves and sexual risk.

Our finding that landlord-related forced moves are associated with HIV sexual risk builds on prior work examining the impact of eviction on HIV risk and/or poor HIV outcomes. For example, in a prior ecological study conducted by our team, county-level eviction rates were associated with county-level rates of both chlamydia and gonorrhea [12]. A prospective cohort study with drug-using Canadian youth also found eviction to be associated with increased risk of syringe sharing [8]. Finally, and similarly, another prospective cohort study in Vancouver with people who inject drugs (PWID) found residential eviction to be associated with increased risk of violence, increased odds of methamphetamine initiation or relapse, and increased odds of detectable HIV-1 RNA viral load (among HIV positive PWID) [5–7].

There are several limitations to the current analyses. First, the cross-sectional design limits our ability to infer a causal relationship between landlord-related forced moves and HIV risk. While the exposure to landlord-related forced moves covered the prior two years and the sexual risk behaviors were reported for the past six months, it is possible that in some cases, the sexual risk behavior preceded the landlord-related forced move. Future longitudinal studies are needed to ascertain the temporal relationship and the pathways through which landlord-related forced moves impact sexual risk. Additionally, and relatedly, future longitudinal studies should examine whether the same issues that cause the landlord-related forced move, such as severe drug problems, also drive sexual risk.

Second, some items in our measurement of landlord-related forced moves did not specifically reference the landlord (e.g., I was forced to move because I was accused of illegal drug activities). However, we included these due to the high likelihood that these items are landlord related [17]. Furthermore, sensitivity analysis indicated the findings were consistent even with the exclusion of these items in the creation of the landlord-related forced move variable. Nonetheless, qualitative research can help to better understand low-income individuals’ experiences with landlord-related forced moves.

Third, our self-reported measures of sexual behavior are imperfect markers of HIV risk. For example, unsafe sex is only risky in terms of HIV acquisition within specific relationship contexts. For example, with regards to HIV, unprotected sex is not risky in a fully monogamous relationship in which both partners are HIV negative. Additionally, our measure of partner concurrency is based on respondents’ assessment of their partners’ behavior and may either under or over report partner concurrency. Of note, in longitudinal qualitative interviews with our participants, participants were able to recognize when their partner was engaging in other sexual relationships. Despite these limitations, unsafe sex and perceived partner concurrency have each been associated with HIV and/or STI acquisition across numerous studies (e.g., [27, 32–34] and are both common measures of HIV risk in the absence of costly seroincidence data. Moreover, a major strength of our findings is the consistency of the associations observed across multiple individual markers of HIV sexual risk using our HIV sexual risk composite. Nonetheless, future studies might examine whether the association between landlord-related forced moves and HIV sexual risk varies within specific relationship contexts.

By focusing on landlord-related forced moves as a specific form of housing instability, while simultaneously expanding the definition of eviction to include landlord-related forced moves, our study contributes to understanding the potential impacts of housing instability on HIV sexual risk. Most of the extant research on forced moves to date focuses on legal eviction even though there are multiple other ways in which landlords may force tenants out before undertaking a legal eviction. Further, most research exploring the relationship between housing and HIV/AIDS in greater depth has focused on unstable housing more broadly [36–40], rather than on the impacts of evictions and other landlord-related forced moves, specifically.

Based on our findings, housing interventions that reduce evictions and/or other landlord-related forced moves may impact HIV sexual risk. Specifically, eviction prevention programs that provide tailored support to individuals and/or households facing a landlord-related forced move may prevent forced moves and decrease HIV sexual risk. Such programs may include tenants-right workshops, advocacy, legal representation, and/or emergency funding [41, 42]. Evidence from studies like ours can be used by eviction prevention leaders and community-based groups to demonstrate the potential health consequences of landlord-related forced moves in their efforts to advocate for protections against forced moves [43]. Finally, in cases where a forced move is unavoidable, interventions might provide individuals and/or households with access to and/or referrals for rapid rehousing, counseling, social services, and other resources.

Of course, while services for those facing housing crises are critical for maintaining existing housing, minimizing the disruption of a forced move, or avoiding homelessness, such efforts do not address one of the largest underlying drivers of landlord-related forced moves. That is, most evictions occur because of non-payment of rent, which is primarily a result of a shortage of affordable housing for low-income Americans [2, 3]. As such, broader policy approaches, such as expanding rental subsidies to offset the severe rent burden that many low-income households face, or addressing zoning policies that place undue burdens on cities to provide affordable housing, may have even more substantial and lasting health impacts.

## Conclusions

Housing is increasingly recognized as a social determinant of health [44]. While a robust body of literature documents the negative impacts of homelessness on health outcomes, there is less research documenting the negative impacts of other forms of unstable housing on physical and mental health outcomes, including HIV acquisition. We have provided evidence that one type of housing instability—landlord-related forced moves—is associated with HIV sexual risk. The current strategic plan to Ending AIDS in America by 2030 focuses primarily on biomedical approaches to prevent, diagnose and treat HIV [45]; however, the potential impact of policy and/or other structural interventions [46], such as those which reduce landlord-related forced moves and/or promote access to stable and affordable housing, should not be overlooked.
